# Supplementary material for: Reservoirs and transmission routes of leprosy; A systematic review
Source: PLoS Negl Trop Dis. 2020 Apr 27;14(4):e0008276. doi: 10.1371/journal.pntd.0008276 (PMC7205316; doi:10.1371/journal.pntd.0008276)
Supplement: S1 Text — (DOCX) [file pntd.0008276.s002.docx]

Animal

1. Sharma R, Singh P, Pena M, Subramanian R, Chouljenko V, Kim J, et al. Differential growth of Mycobacterium leprae strains (SNP genotypes) in armadillos. Infect Genet Evol. 2018;62:20–6.
   No information on prevalence. SNP genotypes in armadillos beyond the scope of this review.
2. Convit J, Aranzazu N, Pinardi ME. Experimental leprosy in Dasypus sabanicola. Int J Lepr. 1979;47(2 supp):338.
   Snowballing, but not wildlife. Susceptibility of other armadillo species experimentally.
3. Walsh GP, Gormus BJ, Meyers WM, Hubbard GB, Baskin GB. Naturally Acquired and Experimental Leprosy in Nonhuman Primates. Am J Trop Med Hyg. 1991 Apr 1;44(4_Part_2):24–7.
   Review.
4. WADE HW. MYCOBACTERIOSIS OF CATS; CAT LEPROSY. Int J Lepr. 1964;32(4):428–9.
   Cat leprosy, but no way to link to human leprosy experimentally.
5. Gee BR, Schiefer B, Ward GE. Letter: Disease resembling feline leprosy. Can Vet J = La Rev Vet Can. 1975;16(1):30.
   Cat leprosy, but no way to link to human leprosy experimentally.
6. Klingmüller G, Sobich E. [Transmission of human leprosy bacteria to hedgehogs]. Naturwissenschaften [Internet]. 1977 Dec [cited 2019 Jun 14];64(12):645–6.
   Experimental unsuccessful attempt.
7. Stinear TP, Brosch R. Leprosy in red squirrels. Science (80- ). 2016;354(6313):702–3.
   Snowballing: not the initial report.
   Not the initial report of leprosy in squirrels.
8. Gormus BJ, Murphey-Corb M, Baskin GB, Uherka K, Martin LN, Marx PA, et al. Interactions between Mycobacterium leprae and simian immunodeficiency virus (SIV) in rhesus monkeys. J Med Primatol. 2000;29(3–4):259–67.
   A hypothesis beyond the scope of this review.
9. Shepard CC. IMMUNOLOGICAL IDENTIFICATION OF FOOT-PAD ISOLATES AS MYCOBACTERIUM LEPRAE BY LEPROMIN REACTIVITY IN LEPROSY PATIENTS. J Exp Med. 1963 Aug 1;118(2):195–204.
   Not the initial report of experimental leprosy in the mouse footpad.
10. da Silva MB, Portela JM, Li W, Jackson M, Gonzalez-Juarrero M, Hidalgo AS, et al. Evidence of zoonotic leprosy in Pará, Brazilian Amazon, and risks associated with human contact or consumption of armadillos. PLoS Negl Trop Dis. 2018;12(6):e0006532.
    Not about wildlife prevalence.
11. Leiker DL, Poelma FG. On the etiology of cat leprosy. IntJLeprOther MycobactDis. 1974;42(3):312–5.
    Outdated.
12. Oktaria S, Effendi EH, Indriatmi W, van Hees CLM, Thio HB, Sjamsoe-Daili ES. Soil-transmitted helminth infections and leprosy: a cross-sectional study of the association between two major neglected tropical diseases in Indonesia. BMC Infect Dis. 2016;16(1):258.
    Not about wildlife prevalence.
13. Rojas-Espinosa O, Løvik M. Mycobacterium leprae and Mycobacterium lepraemurium infections in domestic and wild animals. Rev Sci Tech. 2001;20(1):219–51.
    Review.
14. Fukunishi Y, Meyers WM, Binford CH, Walsh GP, Johnson FB, Gerone PJ, et al. Electron microscopic study of leprosy in a mangabey monkey (natural infection). Int J Lepr other Mycobact Dis. 1984;52(2):203–7.
    Not the initial report of this animal.
15. Arrazuria R, Juste RA, Elguezabal N. Mycobacterial Infections in Rabbits: From the Wild to the Laboratory. Transbound Emerg Dis [Internet]. 2017 Aug 1 [cited 2019 Feb 18];64(4):1045–58. Available from: <http://doi.wiley.com/10.1111/tbed.12474>
    Not related to *M. leprae*.
16. Lumpkin LR, Cox GF, Wolf JE. Leprosy in five armadillo handlers. J Am Acad Dermatol [Internet]. 1983 Dec [cited 2019 Jun 14];9(6):899–903. Available from: <https://pdf.sciencedirectassets.com/272892/1-s2.0-S0190962205X70542/1-s2.0-S0190962283702069/main.pdf?x-amz-security-token=AgoJb3JpZ2luX2VjEMz%2F%2F%2F%2F%2F%2F%2F%2F%2F%2FwEaCXVzLWVhc3QtMSJGMEQCIEuo%2FjatYeZtVr2uOREEVf%2B0UY%2FIce3yP3WMpasudwYEAiAPaXrmTh>
    Not about wildlife prevalence.
17. Filice GA, Greenberg RN, Fraser DW, Greenberg RN, Fraser DW. Lack of observed association between armadillo contact and leprosy in humans. Am J Trop Med Hyg. 1977;26(1):137–9.
    Not about wildlife prevalence.
18. Truman R. Leprosy in wild armadillos. Lepr Rev. 2005;76(3):198–208.
    Review.
19. Binford CH. Transmission of Mycobacterium leprae to animals. Nerve involvement in the ears of hamsters. Int J Lepr. 1965;33(4):865–74.
    Unsuccessful experimental attempt of transmission to an animal species.
20. Silva DA da, Gremião IDF, Menezes RC, Pereira SA, Figueiredo FB, Ferreira RMC, et al. Autochthonous case of feline atypical cutaneous mycobacteriosis in the municipality of Rio de Janeiro-Brazil. Vol. 38, Acta Scientiae Veterinariae. 2018. 327–331 p.
    Unrelated to human leprosy.
21. Convit J, Pinardi ME. Leprosy: confirmation in the armadillo. Science. 1974;184(4142):1191–2.
    Not about wildlife prevalence.
22. WHERRY WB. Mycobacterial infection in birds. II. Leprosy-like disease in the lungs of the Mexican parrot. Int J Lepr. 1952;20(1):108.
    Unrelated to human leprosy.
23. Douglas C. Armadillos and monkeys not the culprits. Aust N Z J Public Health. 2011;35(2):195; author reply 195.
    Opinion.
24. Argaw AT, Shannon EJ, Assefa A, Mikru FS, Mariam BK, Malone JB. A geospatial risk assessment model for leprosy in Ethiopia based on environmental thermal-hydrological regime analysis. Geospat Health. 2006;1(1):105.
    Not about wildlife prevalence.
25. Lane JE, Meyers WM, Walsh DS. Armadillos as a Source of Leprosy Infection in the Southeast. South Med J. 2009;102(1):113–4.
    Reply to article.
26. Thomas DA, Mines JS, Thomas DC, Mack TM, Rea TH. Armadillo Exposure Among Mexican-Born Patients with Lepromatous Leprosy. J Infect Dis. 1987 Dec 1;156(6):990–2.
    Data based on interview with patient.
27. Walgate Robert. Armadillos fight leprosy. Nature. 1981;291(5816):527.
    Related to pathophysiology, not about wildlife prevalence.
28. Walsh GP, Meyers WM, Binford CH, Gerone PJ, Wolf RH, Leininger JR. Leprosy--a zoonosis. Lepr Rev. 1981;52(Suppl 1):77–83.
    Review.
29. Lowy I, Molrine D, Ambrosino D. Genomewide Association Study of Leprosy. N Engl J Med [Internet]. 2010 Apr 15 [cited 2019 Feb 18];362(15):1446–8. Available from: <http://www.nejm.org/doi/abs/10.1056/NEJMc1001451>
    Not about wildlife prevalence.
30. Lumpkin LR, Cox GF, Wolf JE. Leprosy in armadillo handlers. J Am Acad Dermatol. 1984;10(6):1073.
    Case-report, no molecular data.
31. Rees RJ, Weddell AG, Palmer E, Pearson JM. Human leprosy in normal mice. Br Med J. 1969;3(5664):216–7.
    Experimental, not about wildlife prevalence. It is beyond the scope of this article to include all species experimentally infected.
32. Sreevatsa, Girdhar BK, Ipe IM, Desikan K V. Can sandflies be the vector for leprosy? Int J Lepr other Mycobact Dis Off organ Int Lepr Assoc. 1992;60(1):94–6.
    Vector experiment.
33. Torii E, Reppas G, Krockenberger M, Fyfe J, O’Brien C, Malik R. Autochthonous feline leprosy caused by Mycobacterium sp. strain Tarwin affecting a cat from the Central Coast of New South Wales. Aust Vet J. 2016;94(8):285–9.
    Unrelated to human leprosy.
34. Clark BM, Murray CK, Longfield RN, Rasnake MS, Horvath LL, Deye GA. Case-control Study of Armadillo Contact and Hansen’s Disease. Am J Trop Med Hyg. 2008;78(6):962–7.
    Zoonosis article.
35. Poelma FG, Leiker DL. Cat leprosy in the Netherlands. Int J Lepr Other Mycobact Dis. 1974;42(3):307–11.
    Unrelated to human leprosy.
36. Shepard CC. Multiplication of Mycobacterium leprae in the foot-pad of the mouse. Int J Lepr. 1962;30(3):291–306.
    Snowballing, not included in the results table.
37. Lane JE, Walsh DS, Meyers WM, Klassen-Fischer MK, Kent DE, Cohen DJ. Borderline tuberculoid leprosy in a woman from the state of Georgia with armadillo exposure. J Am Acad Dermatol [Internet]. 2006 Oct [cited 2019 Jun 11];55(4):714–6. Available from: <https://pdf.sciencedirectassets.com/272892/1-s2.0-S0190962206X02189/1-s2.0-S0190962206006293/main.pdf?x-amz-security-token=AgoJb3JpZ2luX2VjEG4aCXVzLWVhc3QtMSJHMEUCIDDsUzGcuEnXxApj8kRDS7nL1FfA0WVZxeYfryO%2BtrHWAiEA0h62EDvPeOAs3OLm4hbKXqWwBKgWSGJCk3aiuc7Qag>
    Zoonosis case report.
38. Gunn-Moore D, Shaw S. Mycobacterial disease in the cat. In Pract. 1997;19(9):493–501.
    Unrelated to human leprosy.
39. Galletti G, Cavicchi G, Ussia G. Replication of Mycobacterium leprae in hibernating ground squirrels (Citellus tridecemlineatus). Acta Leprol. 1982;52(88):23–31.
    Snowballing, experimental study, not included in results table.
40. Ishii N, Udono T, Fujisawa M, Idani G, Tanigawa K, Miyamura T, et al. [Leprosy in a chimpanzee]. Nihon Hansen Gakkai zasshi. 2011;80(1):29–36.
    Not the original report.
41. Malik R, O’Brien CR, Malik R. Leprosy – we’ve much left to learn, but are looking to squirrels, cows and cats for insights. J Feline Med Surg. 2017;19(9):977–8.
    Snowballing, not an experiment.
42. Housman G, Malukiewicz J, Boere V, Grativol AD, Pereira LCM, Silva I de O e, et al. Validation of qPCR Methods for the Detection of Mycobacterium in New World Animal Reservoirs. PLoS Negl Trop Dis. 2015;9(11):e0004198.
    Method validation, not about wildlife prevalence.
43. O’Brien CR, Malik R, Globan M, Reppas G, McCowan C, Fyfe JA. Feline leprosy due to Mycobacterium lepraemurium : Further clinical and molecular characterisation of 23 previously reported cases and an additional 42 cases. J Feline Med Surg. 2017;19(7):737–46.
    Cat leprosy not related to human leprosy.
44. Hamilton HK, Levis WR, Martiniuk F, Cabrera A, Wolf J. The role of the armadillo and sooty mangabey monkey in human leprosy. Int J Dermatol. 2008;47(6):545–50.
    Not about wildlife prevalence.
45. Scollard DM, Adams LB, Gillis TP, Krahenbuhl JL, Truman RW, Williams DL. The Continuing Challenges of Leprosy. Clin Microbiol Rev. 2006;19(2):338–81.
    Review.
46. Kimura M, Sakamuri RM, Groathouse NA, Rivoire BL, Gingrich D, Krueger-Koplin S, et al. Rapid Variable-Number Tandem-Repeat Genotyping for Mycobacterium leprae Clinical Specimens. J Clin Microbiol. 2009;47(6):1757–66.
    Not about wildlife prevalence.
47. Matsuo Y, Tasaka H, Utsunomiya S. A culturable Mycobacterium isolated from leproma of a leprosy-transmitted armadillo. Repura. 45(2):63–7.
    Not about wildlife prevalence.
48. Balina LM, Valdez RP, de Herrera M, Costa Cordova H, Bellocq J, Garcia N, et al. Experimental reproduction of leprosy in seven-banded armadillos (Dasypus hybridus). Int J Lepr Other Mycobact Dis. 1985;53(4):595–9.
    Snowballing, case-report of experimental infection.
49. Truman RW, Shannon EJ, Hagstad H V., Hugh-Jones ME, Wolff A, Hastings RC. Evaluation of the origin of Mycobacterium leprae infections in the wild armadillo, Dasypus novemcinctus. Am J Trop Med Hyg. 1986 May;35(3):588–93.
    Snowballing, not about wildlife prevalence.
50. Baskin GB, Gormus BJ, Martin LN, Wolf RH, Blanchard JL, Malaty R, et al. EXPERIMENTAL LEPROSY IN AFRICAN GREEN MONKEYS (CERCOPITHECUS AETHIOPS): A MODEL FOR POLYNEURITIC LEPROSY. Vol. 37, Am. J. Trap. Med. Hyg. 1987.
    Necropsy findings.
51. Meyers WM. Letter: Leprosy and armadillos. South Med J. 1976;69(8):1103.
    Not about wildlife prevalence.
52. Courtin F, Huerre M, Fyfe J, Dumas P, Boschiroli ML. A case of feline leprosy caused by Mycobacterium lepraemurium originating from the island of Kythira (Greece): diagnosis and treatment. J Feline Med Surg. 2007;9(3):238–41.
    Not related to human leprosy.
53. Truman R. Armadillos as a Source of Infection for Leprosy. South Med J. 2008;101(6):581–2.
    Review.
54. Rea TH. Naturally acquired leprosy in animals. West J Med. 1983;138(6):866.
    Review.
55. Bratschi MW, Steinmann P, Wickenden A, Gillis TP. Current knowledge on Mycobacterium leprae transmission: a systematic literature review. Lepr Rev. 2015;86(2):142–55.
    Snowballing, not about wildlife reservoirs or environmental transmission.
56. Rees RJW. Mycobacterial Disease in Man and Animals [Abridged]: Studies on Leprosy Bacilli in Man and Animals. J R Soc Med. 1964;57(6):482–3.
    Review.
57. Suzuki K, Tanigawa K, Kawashima A, Miyamura T, Ishii N. Chimpanzees used for medical research shed light on the pathoetiology of leprosy. Future Microbiol. 2011;6(10):1151–7.
    Review.
58. McDougall AC, Rees RJW, Lowe C. The histopathology of experimental leprosy in the armadillo: Dasypus novemcinctus, Linn., and Dasypus sabanicola, Linn. Int J Lepr. 1979;47(2 Sup.):II 71.
    Report of a histopathological study of experimental infection.
59. Walsh GP, Meyers WM, Binford CH. Naturally acquired leprosy in the nine-banded armadillo: a decade of experience 1975-1985. J Leukoc Biol. 1986;40(5):645–56.
    Review
60. Gunn-Moore DA. Feline mycobacterial infections. Vet J. 2014;201:230–8.
    Review, not related to human leprosy.
61. O’Brien CR, Malik R, Globan M, Reppas G, McCowan C, Fyfe JA. Feline leprosy due to Candidatus ‘Mycobacterium tarwinense’: Further clinical and molecular characterisation of 15 previously reported cases and an additional 27 cases. J Feline Med Surg. 2017;19(5):498–512.
    Not related to human leprosy.
62. Truman RW, Singh P, Sharma R, Busso P, Rougemont J, Paniz-Mondolfi A, et al. Probable Zoonotic Leprosy in the Southern United States. N Engl J Med. 2011;364(17):1626–33.
    Zoonosis, not wildlife prevalence.
63. Deps PD, Alves BL, Gripp CG, Aragao RL, Guedes B, Filho JB, et al. Contact with armadillos increases the risk of leprosy in Brazil: A case control study. Indian J Dermatol Venereol Leprol. 2008;74(4):338–42.
    Zoonosis, not wildlife prevalence.
64. da Silva Ferreira J, Souza Oliveira DA, Santos JP, Ribeiro CCDU, Baêta BA, Teixeira RC, et al. Ticks as potential vectors of Mycobacterium leprae: Use of tick cell lines to culture the bacilli and generate transgenic strains. PLoS Negl Trop Dis. 2018;12(12):e0007001.
    Vector, not wildlife prevalence.
65. Neumann A da S, Dias F de A, Ferreira J da S, Fontes ANB, Rosa PS, Macedo RE, et al. Experimental Infection of Rhodnius prolixus (Hemiptera, Triatominae) with Mycobacterium leprae Indicates Potential for Leprosy Transmission. Lanz-Mendoza H, editor. PLoS One [Internet]. 2016 May 20;11(5):e0156037. Available from: <http://dx.plos.org/10.1371/journal.pone.0156037>
    Vector, not wildlife prevalence.
66. Wheat WH, Casali AL, Thomas V, Spencer JS, Lahiri R, Williams DL, et al. Long-term Survival and Virulence of Mycobacterium leprae in Amoebal Cysts. Vinetz JM, editor. PLoS Negl Trop Dis [Internet]. 2014 Dec 18;8(12):e3405. Available from: <https://dx.plos.org/10.1371/journal.pntd.0003405>
    Vector, not wildlife prevalence.
67. Lahiri R, Krahenbuhl JL. The role of free-living pathogenic amoeba in the transmission of leprosy: a proof of principle. Lepr Rev. 2008;79(4):401–9.
    Vector, not wildlife prevalence.

Environment

1. Akama T, Kawashima A, Tanigawa K, Hayashi M, Ishido Y, Luo Y, et al. Comprehensive Analysis of Prokaryotes in Environmental Water Using DNA Microarray Analysis and Whole Genome Amplification. Pathogens. 2013;2(4):591–605.
   No relevant information on *M. leprae* or *M. lepromatosis.*
2. Bakker MI, May L, Hatta M, Kwenang A, Klatser PR, Oskam L, et al. Genetic, household and spatial clustering of leprosy on an island in Indonesia: a population-based study. BMC Med Genet. 2005;6(1):40.
   Human spatial clustering study, not environmental.
3. Bakker MI, Hatta M, Kwenang A, Faber WR, Van Beers SM, Klatser PR, et al. Population survey to determine risk factors for Mycobacterium leprae transmission and infection. Int J Epidemiol. 2004 Dec;33(6):1329–36.
   Human seroprevalence survey.
4. Ferreira FR, Nascimento LFC. Spatial approach of leprosy in the State of São Paulo, 2009-2012. An Bras Dermatol [Internet]. 2019 Feb [cited 2019 Jun 15];94(1):37–41. Available from: <http://dx.doi.org/10.1590/abd1806-4841.20197351>
   Not about the environment.
5. Paling S, Wahyuni R, Winarni D, Astari L, Adriaty D, Agusni I, et al. Acanthamoeba SP.S-11 phagocytotic activity on Mycobacterium leprae in different nutrient conditions. African J Infect Dis. 2018;12(S):44–8.
   Vector, not environment.
6. Kato L. Autotrophism of an “intracellular parasite” Mycobacterium leprae. Can J Public Health. 1973 Oct;64(Suppl):42–6.
   Not environment.
7. Chaves EC, Costa SV, Flores RL dos R, Neves EOS das, Chaves EC, Costa SV, et al. Índice de carência social e hanseníase no estado do Pará em 2013: análise espacial. Epidemiol e Serviços Saúde. 2017;26(4):807–16. Hagge DA, Parajuli P, Kunwar CB, Rana DRSJB, Thapa R, Neupane KD, et al. Opening a Can of Worms: Leprosy Reactions and Complicit Soil-Transmitted Helminths. EBioMedicine. 2017;23:119–24.
   Not environment.
8. Drancourt M. Looking in amoebae as a source of mycobacteria. Microb Pathog. 2014;77:119–24.
   Review.
9. Duarte-Cunha M, Almeida AS de, Cunha GM da, Souza-Santos R. Geographic weighted regression: applicability to epidemiological studies of leprosy. Rev Soc Bras Med Trop [Internet]. 2016 Feb [cited 2019 Jun 15];49(1):74–82. Available from: <http://dx.doi.org/10.1590/0037-8682-0307-2015>
   Not environment.
10. Nicchio MVC, Araujo S, Martins LC, Pinheiro A V, Pereira DC, Borges A, et al. Spatial and temporal epidemiology of Mycobacterium leprae infection among leprosy patients and household contacts of an endemic region in Southeast Brazil. Acta Trop [Internet]. 2016 [cited 2019 Jun 15];163:38–45. Available from: <http://dx.doi.org/10.1016/j.actatropica.2016.07.019>
    Not environment.
11. 1Blake LA, West BC, Lary CH, Fowler MER, Todd JR. Earthworms near leprosy patients’ homes are negative for acid-fast bacilli by fite stain, providing no link between leprous armadillos (Dasypus novemcinctus) and human leprosy. Microb Ecol. 1989;17(1):105–10.
    Vector, not invironment.
12. Truman RW, Job CK, Hastings RC, Kumaresan JA, Mcdonough CM. Seasonal and spatial trends in the detectability of leprosy in wild armadillos. Epidemiol Infect. 1991;106(3):549–60.
    Beyond the scope if this review.
13. Wheat WH, Casali AL, Thomas V, Spencer JS, Lahiri R, Williams DL, et al. Long-term Survival and Virulence of Mycobacterium leprae in Amoebal Cysts. Vinetz JM, editor. PLoS Negl Trop Dis [Internet]. 2014 Dec 18;8(12):e3405. Available from: <https://dx.plos.org/10.1371/journal.pntd.0003405>
    Vector, not environment.

Vector & zoonosis

1. Skinsnes OK. Editorial: Coughing, sneezing and mosquitoes in the transmission of leprosy. Int J Lepr Other Mycobact Dis. 1975;43(4):378–81.
   Editorial, no new data.
2. Greenwald JS, Domozych R. Armadillos: An emerging zoonosis in Florida. JAAD Case Reports [Internet]. 2017 Jul 1 [cited 2019 May 17];3(4):371. Available from: <https://www.sciencedirect.com/science/article/pii/S2352512617300966?via%3Dihub>
   Reply to paper.
3. Walsh GP, Meyers WM, Binford CH. Indigenous leprosy in armadillos (Dasypus novemcinctus). Int J Lepr. 1979;47(2 Supp):342.
   Conference summary.
4. Rojas-Espinosa O, Løvik M. Mycobacterium leprae and Mycobacterium lepraemurium infections in domestic and wild animals. Rev Sci Tech. 2001;20(1):219–51.
   Experimental infections, review.
5. Lane JE, Meyers WM, Walsh DS. Armadillos as a Source of Leprosy Infection in the Southeast. South Med J. 2009;102(1):113–4.
   Letter to the editor
6. Benchimol JL, Sá MR, Benchimol JL, Sá R, Romero Sa M. Adolpho Lutz and controversies over the transmission of leprosy by mosquitoes. Hist Cienc Saude Manguinhos. 2003;10(Suppl 1):49–93.
   Biography.
7. Walsh GP, Meyers WM, Binford CH, Gerone PJ, Wolf RH, Leininger JR. Leprosy--a zoonosis. Lepr Rev. 1981;52(Suppl 1):77–83.
   Symposium report.
8. Truman R. Armadillos as a Source of Infection for Leprosy. South Med J. 2008;101(6):581–2.
   Review.
9. Walsh GP, Meyers WM, Binford CH. Naturally acquired leprosy in the nine-banded armadillo: a decade of experience 1975-1985. J Leukoc Biol. 1986;40(5):645–56.
   Review.
10. Gormus BJ, Wolf RH, Baskin GB, Ohkawa S, Gerone PJ, Walsh GP, et al. A second sooty mangabey monkey with naturally acquired leprosy: first reported possible monkey-to-monkey transmission. Int J Lepr Other Mycobact Dis. 1988;56(1):61–5.
    Transmission between animals. Reported under animal, not zoonosis or vector.
11. Sharma R, Singh P, Pena M, Subramanian R, Chouljenko V, Kim J, et al. Differential growth of Mycobacterium leprae strains (SNP genotypes) in armadillos. Infect Genet Evol. 2018;62:20–6.
    Not zoonosis or vector: strain variation study.
12. Cleary LC, Suraj S, Haburchak D, Turrentine JE. The Armadillo Factor: Lepromatous Leprosy. Am J Med. 2017;130(10):1163–6.
    Dermatology case-report. No data on zoonosis.
13. Hubbard GB, Lee DR, Eichberg JW, Gormus BJ, Xu K, Meyers WM. Spontaneous Leprosy in a Chimpanzee ( Pan troglodytes ). Vet Pathol. 1991;28(6):546–8.
    Not zoonosis.
14. Deem SL, Noss AJ, Fiorello C V., Manharth AL, Robbins RG, Karesh, William B.rmadillos in the Gran Chaco B. Health Assessment of Free-Ranging Three-Banded (Tolypeutes matacus) and Nine-Banded (Dasypus novemcinctus) Armadillos in the Gran Chaco, Bolivia. J Zoo Wildl Med. 2009;40(2):245–56.
    Animal, not zoonosis or vector.
15. Blake LA, West BC, Lary CH, Todd JR. Environmental nonhuman sources of leprosy. Rev Infect Dis. 1987;9(3):562–77.
    Environmental review, not zoonosis or vector.
16. Hamilton HK, Levis WR, Martiniuk F, Cabrera A, Wolf J. The role of the armadillo and sooty mangabey monkey in human leprosy. Int J Dermatol. 2008;47(6):545–50.
    Review on zoonosis.
17. Truman RW, Shannon EJ, Hagstad H V., Hugh-Jones ME, Wolff A, Hastings RC. Evaluation of the origin of Mycobacterium leprae infections in the wild armadillo, Dasypus novemcinctus. Am J Trop Med Hyg. 1986 May;35(3):588–93.
    Snowballing: doubts on the origin of infection. Not a results of the systematic review.
18. Bratschi MW, Steinmann P, Wickenden A, Gillis TP. Current knowledge on Mycobacterium leprae transmission: a systematic literature review. Lepr Rev. 2015;86(2):142–55.
    Zoonosis only taken into account as armadillo.
19. Truman R, Fine PEMM. “Environmental” sources of Mycobacterium leprae: issues and evidence. Lepr Rev. 2010;81(2):89–95.
    Editorial.
