# Supplementary material for: Reservoirs and transmission routes of leprosy; A systematic review
Source: PLoS Negl Trop Dis. 2020 Apr 27;14(4):e0008276. doi: 10.1371/journal.pntd.0008276 (PMC7205316; doi:10.1371/journal.pntd.0008276)
Supplement: Attachment — Submitted filename: Rebuttal18maart2020.docx [file pntd.0008276.s003.docx]

Dear editor and reviewers,

First of all, we would like to thank all the reviewers for the close reading of the manuscript and the most fruitful comments, suggested corrections/wordings and additional references! Most of the suggestions as you will see have been used to improve the quality of the manuscript.

Reaction to Reviewers' comments on manuscript "Reservoirs and Transmission Routes of Leprosy”

*Responses are displayed in italic text.*

## Reviewer #1:

Objectives/results

This is a review article that utilises a standard literature review PRISMA approach which is appropriate. The objective of providing an overview of worldwise leprosy research regarding reservoirs and transmission is stated at the end of the introduction. The search criteria and inclusion /exclusion process are adequately described, as is how the data were analysed. As a review article, the results comprise summaries and some synthesis of published studies, as stated in the objective The Figures and tables are clear and useful/essential

There are no ethical or regulatory concerns

Follows the first series of comments of reviewer 1 and our responses:

1.	p6 it is unclear to me what the meaning and significance of the sentence " No review protocol was drafted nor registered in advance of this study" is, please clarify.

*This is an adequate remark, we propose the following alternative sentence: The review protocol was not pre-registered at PROSPERO.*

2.	PRISMA acronym definition and reference should be included in the text p6

*This has been added.*

3.	p6 It is unclear where the additional 57 articles found through snowballing are included in Figure 1, from the text it reads as if there are 80 +57 but this is not reflected clearly in Figure 1

*This has been edited accordingly in the figure and we have adjusted the number of both articles groups following additional suggestions by the reviewers.*

4.	" A discussion of the quality of methods has been incorporated" needs further qualification, as this was not done for all studies and how 'quality' is asssessed is unclear. Suggest reword along the lines of ' discussion of the limitations of some studies is incorporated where these could have influenced the findIngs or conclusions made....'

*This has been rephrased accordingly.*

Conclusion/Discussion/Abstract:

5.	It is not made clear to the reader whether the suggestion of grouping pathogens causing leprosy-like diseases into an *M.leprae*-related complex in research and documentation is proposed and recommended for the first time here by the authors or whether this reflects this usage previous research and publications; if so this should be clearly stated as a new proposal.

*This has been edited throughout the manuscript to be in accordance with the fact that this has been proposed in: Editorial. Leprosy – we’ve much left to learn, but are looking to squirrels, cows and cats for insights. J Feline Med Surg. 2017;19(9):977–8. (new reference 144).*

Textual suggestions/corrections

6. p5 Introduction

last paragraph states that it is a review regarding 'reservoirs and transmission' but it does not actually review direct human to human transmission and so this should be made clearer.

*Edited accordingly*

Synopsis.M.leprae and M. lepromatosis both target skin and peripheral nervous systems, not just M.leprae.

line 6 insert 'created by' in front of 'coughing and sneezing..'

*Edited accordingly*

p2 line 3 M.leprae and M. lepromatosis both target skin and peripheral nervous systems, not just M.leprae line 5. ' selective prevalence' is not the correct term in my opinion to describe local/geographocal differences in prevlence, as it implies the pathogen 'selects' where it causes disease. An alternative term such as 'spatial inequaility' could be used to describe the endemic pockets.

*Edited accordingly*

line 11 insert 'created by' in front of 'coughing and sneezing..'

*Edited accordingly*

line 18, you state the two bacilli being 'found' in red squirresl - in fact the DNA of these two bacilli was detected - and it should also be stated that the disease leprosy was also found in this species

*Edited accordingly*

line 28. Suggest change the phrase ( and in the conclusion where you use this same wording)" Reducing transmission cannot be expected from the human healthcare domain alone..... to something like " A reduction in transmission cannot be expected to be accomplished by actions or interventions from the human healthcare domain alone,..."

*Edited accordingly*

Introduction

p4 line 6: insert 'created by' in front of 'coughing and sneezing..'

*Edited accordingly*

p4 end of 2nd pargrah' ' in cases of leprosy'

*Edited accordingly*

p 5 line 1 see comment above re the term selective prevalence p 5 third paragraph add that the disease leprosy was also found in red squirrels as well as the bacterial DNA detected

*Edited accordingly*

Method

p 6 penultimate line, 'Data..... were' ( not was)

*Edited accordingly*

Results

p8 line 1 change ' symptoms' to ' clinical signs' - animals cannot have symptoms, only humans, it is not a correct veterinary term

*Edited accordingly*

p8 second paragraph 2nd sentence Do not start a sentence with ' But also...'

*Edited accordingly*

p16 'Siberian chipmuck ( Tamias sibiricus n=35) ' is duplicated

*Edited accordingly*

p17 some common species names are wrong, although I have not checked the original text it would be more useful to give the more usual English common name; Procyon cancrivoras is the crab-eating or South American raccoon, not sure what' hand-skinned' means?. Gallictis vittata is the greater grison, not ferret; Cerdyon thous is the crab-eating fox.

*Edited accordingly*

Discussion

p27 line 4 change "English red squirrels ' to British and Irish re squirrels' and see my abocve comments regarding reservoirs definition - as you do state in the abstract it should be stated throughout that animals and environment are possible reservoirs only.

*Edited accordingly*

Summary and General Comments

This is a useful systematic review article providing an overview of global leprosy research , using historical and new research data on leprosy transmission to summarise the current state of knowledge on possible reservoirs and possible transmission routes, to highlight wildlife and environmental reservoirs ( including the recently discovered red squirrel) and encouraging a One Health approach in light of this to future leprosy resreach and interventions.

It is generally well written but it is clear that English is not first language of the authors and there are numerous grammatical errors and some awkward phrasing throughout, some of which I have specified , eg ' data' is plural not singular - but a further thorough review by a native english speaker is recommended.

*We have performed an additional round of English corrections*

The use of the term reservoir is made throughout. I would recommed a definition of this term is given and some discussion of this integrated, as this is an important concept of great relevance to this manuscript. Recommed the definition by Viana et al (2014): A 'reservoir of infection' is defined with respect to a target population as 'one or more epidemiologically connected populations or environments in which a pathogen can be permanently maintained and from which infection is transmitted to the target population' <https://www.ncbi.nlm.nih.gov/pmc/articles/PMC4007595/>

*We are most grateful for this excellent textual suggestion and reference, that have been included in the introduction.*

## Reviewer #2:

Objectives/results

The authors present here a scholarly meta-analysis of the literature concerning environmental sources of leprosy bacilli. They are uniquely qualified senior investigators with well recognized expertise in medicine, leprosy, medical history as well as veterinary medical sciences.

Widespread availability of effective drug therapy has markedly diminished the prevalence of leprosy over that last 30 years; but the new case detection rate has remained low and steady over the last decade. Though traditionally considered a disease transmitted between humans, long-term steady prevalence raises the likelihood that sources other than infected humans are involved in leprosy transmission and play important roles in perpetuating the modern infection. To better understand this issue the authors have made a systematic review of the literature concerning environmental sources of leprosy bacilli. They clearly describe their approach to gather and review the literature, which included thousands of pre-selected articles, and their strategy to organize and focus their review on select papers. This is the most thorough review of environmental sources of leprosy bacilli in the last 40 years.

The results of their literature searches are thoroughly documented and sorted to different topic areas. The 2 accompanying figures clearly detail their process and the scope of their review.

*We would like to thank reviewer 2 for his honorable compliments.*

Conclusions

The authors conclude that the literature is replete with examples of leprosy bacilli being recovered from the environment and that the body of knowledge concerning this issue is expanding in such a manner as the topic can no longer be considered anecdotal. They offer that further reduction in leprosy transmission cannot be expected from the human healthcare domain alone, as the disease cycle and its ecology is likely far too complex. A better understanding of the possible roles of animal or environmental reservoirs is needed. To achieve this, they suggest a paradigm shifting One Health transdisciplinary research approach incorporating the skills of scientists beyond those traditionally involved in leprosy research, and further integrating leprosy with other neglected tropical diseases.

*Ibid*

Editorial comment suggestions:

An important area not mentioned is that clarifying the importance of environmental reservoirs can also have important direct impact on patients. The stigma and anxiety they experience after a diagnosis of leprosy is often exacerbated by their lack of knowledge about how or from where they may have acquired this infection. Understanding that leprosy has clear environmental or biological origins can be quite beneficial in ameliorating the social and psychological impacts of leprosy.

*We fully agree with this observation but we decided that adding a statement on this issue is not in line with the scope of this review.*

In modern times much of the literature includes PCR based assays, usually detecting single target segments of M. leprae or M. lepromatosis DNA. Few investigators perform blinded analyses and even fewer include blinded randomized controls in their assay runs. Seldom are internal control standards like those normally used in clinical laboratories incorporated into studies performed in research laboratories. This lack of rigor contributes to the anecdotal assessment sometimes given to molecular studies reporting M. leprae in non-human sources. The authors should acknowledge the lack of standard controls in many studies and encourage others to exercise greater rigor in environmental studies of Ml and MLPM.

Similarly, multiple authors suggest that mere detection of M. leprae 16s RNA is indicative of viability of the organisms. However, 16s is produced in massive quantities in bacilli and may have a half-life as long as DNA itself. I am not aware of any literature assessing what level of 16s detection is actually associable with viability of M. leprae -- 16s concentration can be seen to rise and fall during growth or decline of leprosy bacilli, but static detection of 16s is no more meaningful than detecting DNA. Some comment about the lack of a good standard for environmental viability would be useful to include.

*We have used the arguments of reviewer 1 to strengthen the paragraph on standardization issues in the discussion.*

Minor items:

The methods section refers to papers “excluded”. The Supplementary data section refers to papers “rejected”. Please make consistent and add an explanation to the lists in the supplementary sections.

*Edited accordingly*

Page 11 last paragraph line 12: I am pretty sure you mean to say “did NOT grow on Lowenstein-Hensen.

*Edited accordingly*

Page 22 next to last line: should be …settlers ‘since’ the 16th century.

*Edited accordingly*

Page 24: Please comment on viability as above

*Included accordingly*

## Reviewer #3:

Reviewer #3: The manuscript by Ploemacher, et al., is a review of the current literature on confirmed and possible reservoirs of leprosy bacilli and routes of transmission of leprosy. Although multidrug therapy has reduced the prevalence of leprosy, the number of new cases each year has remained relatively constant. Therefore, the authors argue, human-to-human transmission is not the only way leprosy can be acquired. Animal and environmental reservoirs, as well as vectors, may play a role in transmission of leprosy to humans. The choice of articles included in the review is justified and appropriate. The manuscript is well written and interesting to read. I agree that leprosy transmission is likely much more complicated than we think it is. This is an important area for research. I like the One Health approach.

2. page 4, although the Ziehl-Neelsen stain is of historical value, the authors should mention the Fite stain as it was a vast improvement for detecting M. leprae in tissues.

*Edited accordingly*

3. page 4, regarding phylogenetics, there are currently “…at least five BRANCHES (0-4)” but only 4 SNP types.

*We would like to thank you for this most appropriate correction*! *Edited accordingly*

4. page 11, regarding the study by Amezcua et al., “Homogentates of infected nodules and lymph nodes did NOT grow on Lowenstein-Jensen…”

*Edited accordingly*

5. page 13, line 3 should be “Colombia”

*Corrected accordingly*

6. page 17, second paragraph: should that be SNP type 3K, rather than 3I? 3K is branch 0 7. page 17, regarding the study by Pedrini, et al., “…hand-skinned ‘what’…”? Raccoon?

Edited accordingly

7. page 19, the authors should provide references for their comment regarding the short half-life of RNA and the appropriateness of using RNA expression as an indicator of viability, especially the use of ribosomal RNA. Messenger RNA has a short half-life and can indicate viability, but the use of ribosomal RNA as a viability indicator is controversial.

*We added the required reference!*

9. page 22, regarding the studies by Truman, et al., the concluding sentence is rather strong. They concluded that both armadillos and humans in the area were infected with the same strain of M. leprae and that leprosy may be a zoonosis in the region.

*We have edited accordingly.*

10. page 24, last sentence of first paragraph: “…the fluorescent staining of living bacteria allowed detection of actual bacteria inside the vector” is not clear. Are you stating that only live bacteria fluoresced? Please provide the reference.

*We have edited accordingly.*

11. Tables 1, 2, and 3: The authors present an overview of the methods applied in these papers. One such method is acid fast bacilli (AFB) staining. AFB in the skin, even in a granuloma, is not confirmatory of leprosy; however, AFB in a nerve is. It would greatly improve the manuscript and be much more informative to the reader if the Tables noted whether or not AFB were observed in a nerve, especially in the older papers before PCR was available. This would also help us understand what kind of reservoir an animal may be, e.g., they actually get leprosy disease or they are carriers and shed it into the environment, etc. It would bolster your One Health approach. Also, growth in mouse footpad is not really informative unless it was confirmed to be M. leprae, so that method could be removed.

*Edited accordingly*

12. page 27, last paragraph regarding the designation of “an M. leprae-related complex in research and documentation.” I think this is too preliminary; however it will generate discussion. Therefore, if the authors want to make this proposal, they should elaborate and expand their reasons in this Discussion. The important question is how does one define a “complex?” Is it based simply on genetic similarities found through sequencing a few genes? Or, should it be based on disease manifestations? Both? As stated above, mycobacterial infection of the skin, even in granulomas, is not necessarily leprosy-like. What makes leprosy the devastating disease that it is is the fact that it invades and damages nerves.

*Thank you for this important remark. We have taken precautions in the text to prevent misinterpretation.*

Additional comment CFP (Associate Editor): Recommend reviewing article:

Unsolved matters in leprosy: a descriptive review and call for further research. Franco-Paredes C, Rodriguez-Morales AJ. Ann Clin Microbiol Antimicrob. 2016 May 21;15(1):33. doi: 10.1186/s12941-016-0149-x. PMID: 27209077. This article of which I am one of the authors of this review has some references on environmental sources of M. leprae and/or M. lepromatosis that I did not see included in your review. I am an not explicitly suggesting to include this article as a reference, its up to you if you find it useful or not.

*We have added the reference and would like to thank you for the suggestion.*
